# The Utilization of Activated Charcoal in the Management of Anaphylaxis: A Case Series

**DOI:** 10.7759/cureus.31949

**Published:** 2022-11-27

**Authors:** Murat Duyan, Nafis Vural

**Affiliations:** 1 Emergency Medicine, Antalya Training and Research Hospital, Antalya, TUR; 2 Emergency Medicine, Ereğli State Hospital, Konya, TUR

**Keywords:** drug-induced anaphylaxis, food-induced anaphylaxis, toxin, activated charcoal, anaphylaxis

## Abstract

Anaphylaxis is a sudden onset of systemic hypersensitivity caused by mast cell and basophil degranulation. Food, Hymenoptera venom, and drug allergy are among the leading causes of anaphylaxis, particularly in adults. We can consider anaphylaxis caused by swallowing food or medication as a form of poisoning. Because in anaphylaxis, just like in poisoning, an allergen entering the body poses a life-threatening risk. Therefore, the allergen should be removed from the digestive system immediately. However, the decontamination of the gastrointestinal tract is not routinely used to prevent further absorption of allergens from the intestine into the systemic circulation. Among the gastrointestinal decontamination methods is the use of activated charcoal. In this article, we present four patients who developed anaphylaxis due to drug and food intake and were administered oral activated charcoal after their primary treatment (on average, 15-45 minutes after the first presentation) was completed. The youngest of the patients was 22 years old, and the oldest was 40. No side effects, prolonged anaphylactic state, and biphasic reactions were observed in the follow-up of the patients. All patients were discharged after 48-72 hours of hospitalization. The routine approach to poisoning treatment includes patient stabilization, toxidrome recognition, antidote administration, and supportive care, as well as measures to enhance toxin elimination. In anaphylaxis caused by oral allergens, the substance that initiates the reaction can be compared to a kind of toxin. Eliminating the allergen and reducing its absorption could be achieved by administering activated charcoal. Activated charcoal should be considered adjunctive therapy in treating food and oral drug-induced anaphylaxis. This treatment, when administered in a timely manner, might prevent the development of biphasic reactions and the prolongation of the allergic process in anaphylaxis.

## Introduction

Anaphylaxis is a sudden onset of systemic hypersensitivity caused by mast cell and basophil degranulation [1]. The incidence of anaphylaxis is increasing worldwide every day [2]. Food, Hymenoptera venom, and pharmaceutical allergies are among the primary triggers of anaphylaxis, notably in adults [3]. Antibiotics, particularly beta-lactams; iodinated contrast agents (ICM); dextrans; allergic extracts; and nonsteroidal anti-inflammatory drugs have been identified as major causes of drug-induced anaphylaxis [4,5]. Peanuts (23.9%), tree nuts (21.6%), shellfish (16.1%), and eggs and milk (both 10.1%) are the most frequent triggers of food allergies [3].

Anaphylaxis emergency care includes appropriate triage, epinephrine administration, and general respiratory and cardiovascular system management [6]. Intramuscular epinephrine is the mainstay of treatment for anaphylaxis. Adjunct medicines consist of antihistamines, corticosteroids, and bronchodilators. When anaphylaxis is resistant to epinephrine, glucagon and other vasopressors should be administered [6]. If an airway obstruction is present, airway management is crucial. We can consider anaphylaxis caused by swallowing food or medication as a form of poisoning. Because in anaphylaxis, just like in poisoning, an allergen entering the body poses a life-threatening risk. Therefore, the allergen should be removed from the digestive system immediately. However, the decontamination of the gastrointestinal tract is not commonly used to restrict allergen absorption through the intestines into the bloodstream. Among the gastrointestinal decontamination methods is the use of activated charcoal. In this case series, we described the use of activated charcoal in treating four patients with anaphylaxis (three drug-induced and one food-induced) and the proposed benefits of this treatment.

## Case presentation

Case 1

A 22-year-old female patient was admitted to the emergency department (ED) with complaints of rash and weakness in the body that started 30 minutes after using a naproxen sodium oral tablet (550 mg) for headaches. The patient had no pertinent medical history or allergies. Upon arrival, her blood pressure was 90/40 mmHg, pulse 130 beats per minute, respiratory rate 25 breaths per minute, pulse oximetry 95% on room air, and temperature 39.6°C. In the patient's physical examination, there was diffuse hyperemia of the skin and minimal edema of the uvula.

Case 2

A 25-year-old female patient without any known comorbidity presented to the ED with complaints of abdominal cramps, nausea, itching in the body, and dizziness 45 minutes after taking diclofenac sodium oral tablet (75 mg) for ankle pain. The patient had a blood pressure of 90/50 mmHg and a pulse rate of 140 beats per minute. Physical examination revealed urticarial plaques on the back and abdomen, abdominal distention, and diffuse abdominal tenderness. The rest of the examination was unremarkable.

Case 3

A 36-year-old male patient presented to the ED with complaints of difficulty swallowing, itching, and dizziness 25 minutes after taking an amoxicillin oral tablet (1000 mg) for acute tonsillitis. The patient has no known history of drug allergies. However, it was learned that he used penicillin-derived drugs for the first time. On physical examination, there was moderate uvular edema, minimal bronchospasm in breath sounds, and hyperemic lesions in the extremities. The patient had a blood pressure of 90/50 mmHg, a pulse rate of 140 beats per minute, and pulse oximetry of 89% on room air. Since severe respiratory and circulatory collapse was observed in the patient, even if it was the first encounter, it was considered anaphylaxis.

Case 4

A 40-year-old male patient with a known strawberry allergy was taken by ambulance to the ED after fainting upon eating strawberries. There is no past medical history. The patient was unconscious when he arrived. The patient's blood pressure was 80/40 mmHg, heart rate was 130/minute, and pulse oximetry was 95%. On physical examination, the patient was unconscious, and diffuse skin urticarial lesions and uvula edema were present.

Activated charcoal (1 mg/kg) was administered orally to these four patients who applied to the emergency department after the first treatment protocol (intramuscular epinephrine, pheniramine maleate, methylprednisolone, and intravenous hydration). This treatment began approximately 15-45 minutes after the initial admission. None of these patients had immediate deterioration, side effects, biphasic reactions to anaphylaxis, or prolonged anaphylactic states during observation. The patients were discharged after 48-72 hours of hospitalization.

## Discussion

In this case series, oral activated charcoal therapy was added to the standard therapies for anaphylaxis brought on by an oral allergen. There were no reported cases of this novel treatment in peer-reviewed literature. Three models of the anaphylactic syndrome have been described: monophasic, biphasic, and persistent. The monophasic type of anaphylaxis, which accounts for 70%-90% of cases, peaks at 30-60 minutes and disappears within an hour without any recurrence of symptoms [7]. The recurrence of symptoms hours after the resolution of the first occasion without re-exposure to the trigger is referred to as biphasic anaphylaxis [8]. In one study, the biphasic type was variably reported to occur in <1%-23% of reactions [9]. Anaphylaxis that lasts for days or weeks is known as protracted or persistent anaphylaxis [7]. Therefore, we propose orally administering activated charcoal to prevent biphasic and persistent anaphylaxis, which may occur by reducing the absorption of allergen substances (food and drug) in the gastrointestinal tract of patients.

The routine approach to poisoning treatment includes patient stabilization, toxidrome recognition, antidote administration, and supportive care, as well as measures to enhance toxin elimination. In anaphylaxis caused by oral allergens, the substance that initiates the reaction can be compared to a kind of toxin. Eliminating this allergen and reducing its absorption might be achieved by the adsorption of this allergen with activated charcoal. Vadas and Perelman showed that activated charcoal rapidly removed both IgE-binding and IgG-binding peanut proteins from the solution at both neutral and acidic pH [10]. These findings imply that administering activated charcoal may be convenient as a supplement to reduce or stop more absorption of peanut protein from the digestive system. In a study by Kopper et al. in a pig animal model, it was revealed that the allergen protein released from peanuts could be bound by activated charcoal in an environment with pH values similar to those in the stomach and intestines [11]. Furthermore, it has been previously determined that activated charcoal administered as a suspension prevents the absorption of various drugs such as diazepam, ibuprofen, citalopram, and fluoxetine [4,12]. All of the available evidence supports the administration of oral activated charcoal in the treatment of allergen-induced anaphylaxis. However, only a case series of four patients was described in this article. Larger randomized controlled studies are needed to obtain more conclusive results.

## Conclusions

Activated charcoal is indispensable for decontamination in the treatment of poisoning. It may be helpful within a specific timeframe before the absorption of the active allergen into the bloodstream. Therefore, activated charcoal should be considered an adjunctive therapy in treating food and oral drug-induced anaphylaxis. This treatment can prevent the development of biphasic reactions and the prolongation of the anaphylactic process in anaphylaxis, but there is a need for further research. In addition, the observation periods in the treatment of anaphylaxis may be shortened.

## References

[1] Giavina-Bianchi P, Aun MV, Kalil J (2018). Drug-induced anaphylaxis: is it an epidemic?. Curr Opin Allergy Clin Immunol.

[2] Turner PJ, Campbell DE, Motosue MS, Campbell RL (2020). Global trends in anaphylaxis epidemiology and clinical implications. J Allergy Clin Immunol Pract.

[3] Gonzalez-Estrada A, Silvers SK, Klein A, Zell K, Wang XF, Lang DM (2017). Epidemiology of anaphylaxis at a tertiary care center: a report of 730 cases. Ann Allergy Asthma Immunol.

[4] Leone R, Conforti A, Venegoni M (2005). Drug-induced anaphylaxis : case/non-case study based on an italian pharmacovigilance database. Drug Saf.

[5] Renaudin JM, Beaudouin E, Ponvert C, Demoly P, Moneret-Vautrin DA (2013). Severe drug-induced anaphylaxis: analysis of 333 cases recorded by the Allergy Vigilance Network from 2002 to 2010. Allergy.

[6] McHugh K, Repanshek Z (2022). Anaphylaxis: emergency department treatment. Emerg Med Clin North Am.

[7] LoVerde D, Iweala OI, Eginli A, Krishnaswamy G (2018). Anaphylaxis. Chest.

[8] Lee S, Sadosty AT, Campbell RL (2016). Update on biphasic anaphylaxis. Curr Opin Allergy Clin Immunol.

[9] Grunau BE, Li J, Yi TW (2014). Incidence of clinically important biphasic reactions in emergency department patients with allergic reactions or anaphylaxis. Ann Emerg Med.

[10] Vadas P, Perelman B (2003). Activated charcoal forms non-IgE binding complexes with peanut proteins. J Allergy Clin Immunol.

[11] Kopper R, Van T, Kim A, Helm R (2011). Release of soluble protein from peanut (Arachis hypogaea, Leguminosae) and its adsorption by activated charcoal. J Agric Food Chem.

[12] Lapatto-Reiniluoto O, Kivistö KT, Neuvonen PJ (1999). Effect of activated charcoal alone or given after gastric lavage in reducing the absorption of diazepam, ibuprofen and citalopram. Br J Clin Pharmacol.

